# Variable degree of mosaicism for tetrasomy 18p in phenotypically discordant monozygotic twins—Diagnostic implications

**DOI:** 10.1002/mgg3.1526

**Published:** 2020-12-14

**Authors:** Małgorzata Rydzanicz, Pawel Olszewski, Darek Kedra, Hanna Davies, Natalia Filipowicz, Bozena Bruhn‐Olszewska, Marco Cavalli, Krzysztof Szczałuba, Marlena Młynek, Marcin M. Machnicki, Piotr Stawiński, Grażyna Kostrzewa, Paweł Krajewski, Dariusz Śladowski, Krystyna Chrzanowska, Jan P. Dumanski, Rafał Płoski

**Affiliations:** ^1^ Department of Medical Genetics Medical University of Warsaw Warsaw Poland; ^2^ Faculty of Pharmacy and 3P Medicine Laboratory International Research Agendas Programme Medical University of Gdańsk Gdańsk Poland; ^3^ Department of Immunology, Genetics and Pathology and Science for Life Laboratory Uppsala University Uppsala Sweden; ^4^ Department of Medical Genetics The Children's Memorial Health Institute Warsaw Poland; ^5^ Department of Immunology Medical University of Warsaw Warsaw Poland; ^6^ Postgraduate School of Molecular Medicine Medical University of Warsaw Warsaw Poland; ^7^ Department of Forensic Medicine Medical University of Warsaw Warsaw Poland; ^8^ Department of Transplantology and Central Tissue Bank Centre for Biostructure Medical University of Warsaw Warsaw Poland

**Keywords:** copy number variants, phenotypically‐discordant monozygotic twins, post‐zygotic mutations, somatic chromosomal mosaicism, tetrasomy 18p, whole‐exome sequencing

## Abstract

**Background:**

Phenotypically discordant monozygotic twins (PDMZTs) offer a unique opportunity to study post‐zygotic genetic variation and provide insights into the linkage between genotype and phenotype. We report a comprehensive analysis of a pair of PDMZTs.

**Methods:**

Dysmorphic features and delayed neuro‐motor development were observed in the proband, whereas her twin sister was phenotypically normal. Four tissues (blood, skin, hair follicles, and buccal mucosa) from both twins were studied using four complementary methods, including whole‐exome sequencing, karyotyping, array CGH, and SNP array.

**Results:**

In the proband, tetrasomy 18p affecting all studied tissues except for blood was identified. Karyotyping of fibroblasts revealed isochromosome 18p [i(18p)] in all metaphases. The corresponding analysis of the phenotypically normal sister surprisingly revealed low‐level mosaicism (5.4%) for i(18p) in fibroblasts.

**Conclusion:**

We emphasize that when mosaicism is suspected, multiple tissues should be studied and we highlight the usefulness of non‐invasive sampling of hair follicles and buccal mucosa as a convenient source of non‐mesoderm‐derived DNA, which complements the analysis of mesoderm using blood. Moreover, low‐level mosaic tetrasomy 18p is well tolerated and such low‐level mosaicism, readily detected by karyotyping, can be missed by other methods. Finally, mosaicism for low‐level tetrasomy 18p might be more common in the general population than it is currently recognized, due to detection limitations.

## Graphical abstract

Herein, for the first time, we present phenotypically discordant monozygotic twins due to variable degree of mosaicism for tetrasomy 18p. In the proband tetrasomy 18p affecting all studied tissues (skin fibroblasts, hair follicles, and buccal mucosa) except for blood was identified. Karyotyping of proband's fibroblasts revealed isochromosome 18p [i(18p)] in all metaphases. The corresponding analysis of the phenotypically normal sister surprisingly revealed low‐level mosaicism (5.4%) for i(18p) in fibroblasts. We emphasize that when mosaicism is suspected, multiple tissues should be studied and low‐level mosaicism, readily detected by karyotyping, can be missed by other methods.

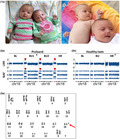

## 1 INTRODUCTION

The somatic or post‐zygotic genetic variation (mosaicism) is understudied and consequently underestimated in the important aspect of human genetics (Forsberg et al., 2017). Mosaic mutations may produce a range of phenotypic manifestations thus being challenging for clinical diagnosis and genetic counseling. Monozygotic twins (MZTs) offer a model for studies of somatic variation, since any genetic differences between MZTs represent an irrefutable example of post‐zygotic mosaicism. Furthermore, when MZTs display genetic differences and are phenotypically discordant, they offer a unique opportunity of linkage between genotype and phenotype (Bruder et al., 2008; Razzaghian et al., 2010). Herein we report a pair of Phenotypically Discordant MZTs (PDMZTs) with mosaicism of 18p tetrasomy. The phenotypically abnormal twin had this aberration in ectodermal tissues (hair follicles and buccal mucosa) and in some mesodermal cells (skin fibroblasts), but not in blood.

## 2 MATERIALS AND METHODS

### 2.1 Ethical compliance

**FIGURE 1:**
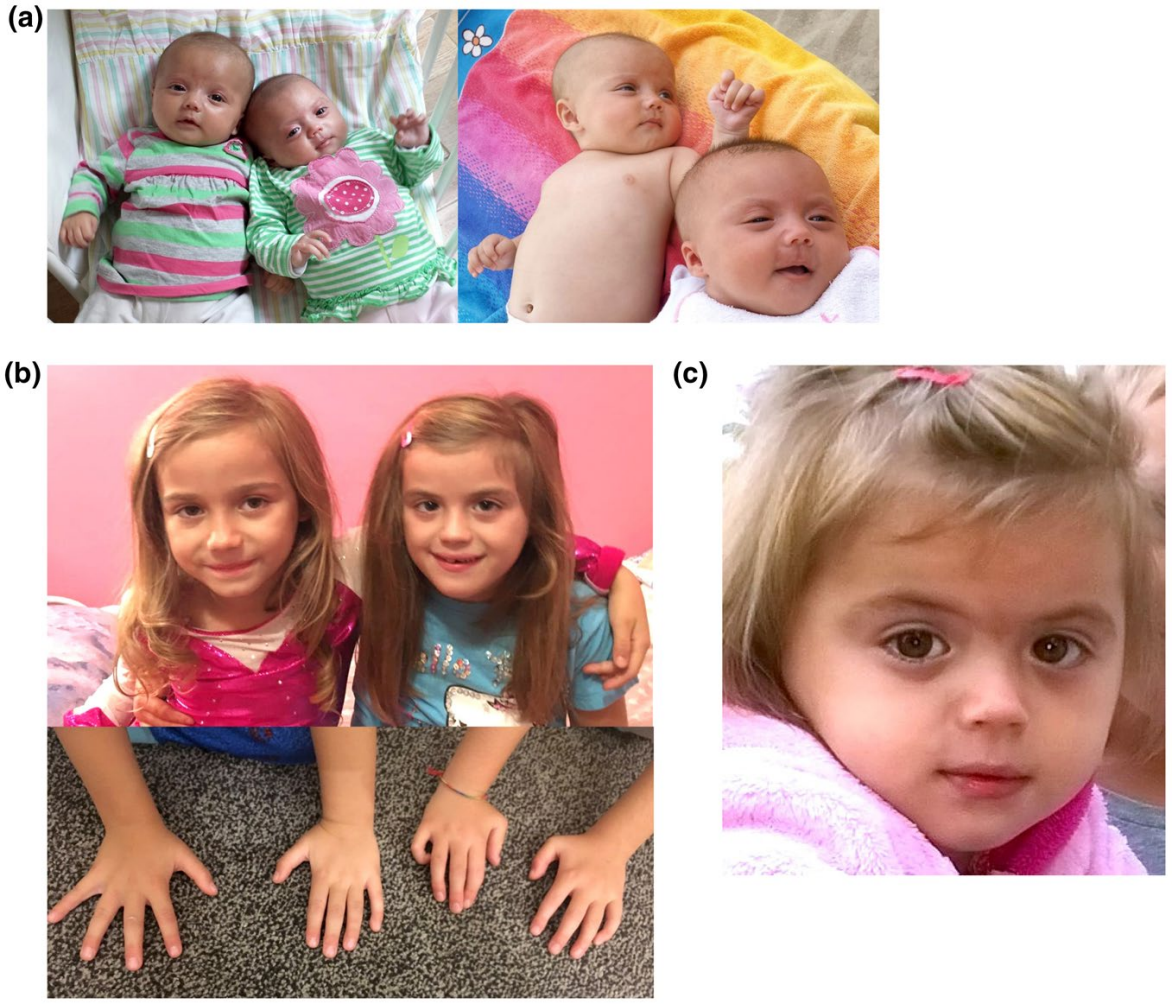
Images of studied phenotypically discordant monozygotic twins at various ages. Panel (a) as neonates and infants (phenotypically normal on the left and phenotypically abnormal on the right), when subtle dysmorphic differences can be noted in the phenotypically abnormal twin, such as triangular, flat face, and thin upper lip. Panel (b) at the age of 6 years (phenotypically normal on the left and phenotypically abnormal on the right) when characteristic flat philtrum can be seen (upper panel) and distal arthrogryposis (lower panel). (c) The face of phenotypically abnormal twin at the age of 4 years

The study protocol was approved by the Ethical Committees of the Medical University of Warsaw (KB/128/2014), Gdansk Medical University (NKBBN/564/2018), and of Uppsala‐region in Sweden (Dnr 2008/270). Written consent was obtained from the parents for the sampling of twins and publication of photographs. Monozygotic twin sisters were born at 37 weeks of gestation after an uneventful pregnancy and delivery by cesarean section. They were daughters to non‐consanguineous parents of Polish and Italian origin. Dysmorphic features and delayed neuro‐motor development were observed in the proband, whereas her twin sister was phenotypically normal (Figure 1, and Table S1).

The family members were sampled independently and studied in parallel at three centers: Medical University of Warsaw, Poland, and 3P Medicine Laboratory established between the Medical University of Gdansk, and Uppsala University in Sweden. The details of the methodology are provided in Supplementary Materials. Briefly, the Warsaw team performed whole‐exome sequencing (WES) on DNA from head‐derived hair follicles. Cultured skin fibroblasts of both twins were studied using metaphase G‐banding karyotyping and DNA extracted from skin fibroblasts was studied using array‐based Comparative Genomic Hybridization (aCGH). Blood, buccal mucosa (separately on both sides of the mouth), and hair follicles (head) as well as blood from both parents were SNP‐genotyped using Global Screening Array (Illumina) and analyzed at 3P Medicine Laboratory as described previously (Razzaghian et al., 2010).

## 3 RESULTS

### 3.1 The phenotypically abnormal twin displayed high‐level mosaicism for i(18p) in several but not all tissues

**FIGURE 2:**
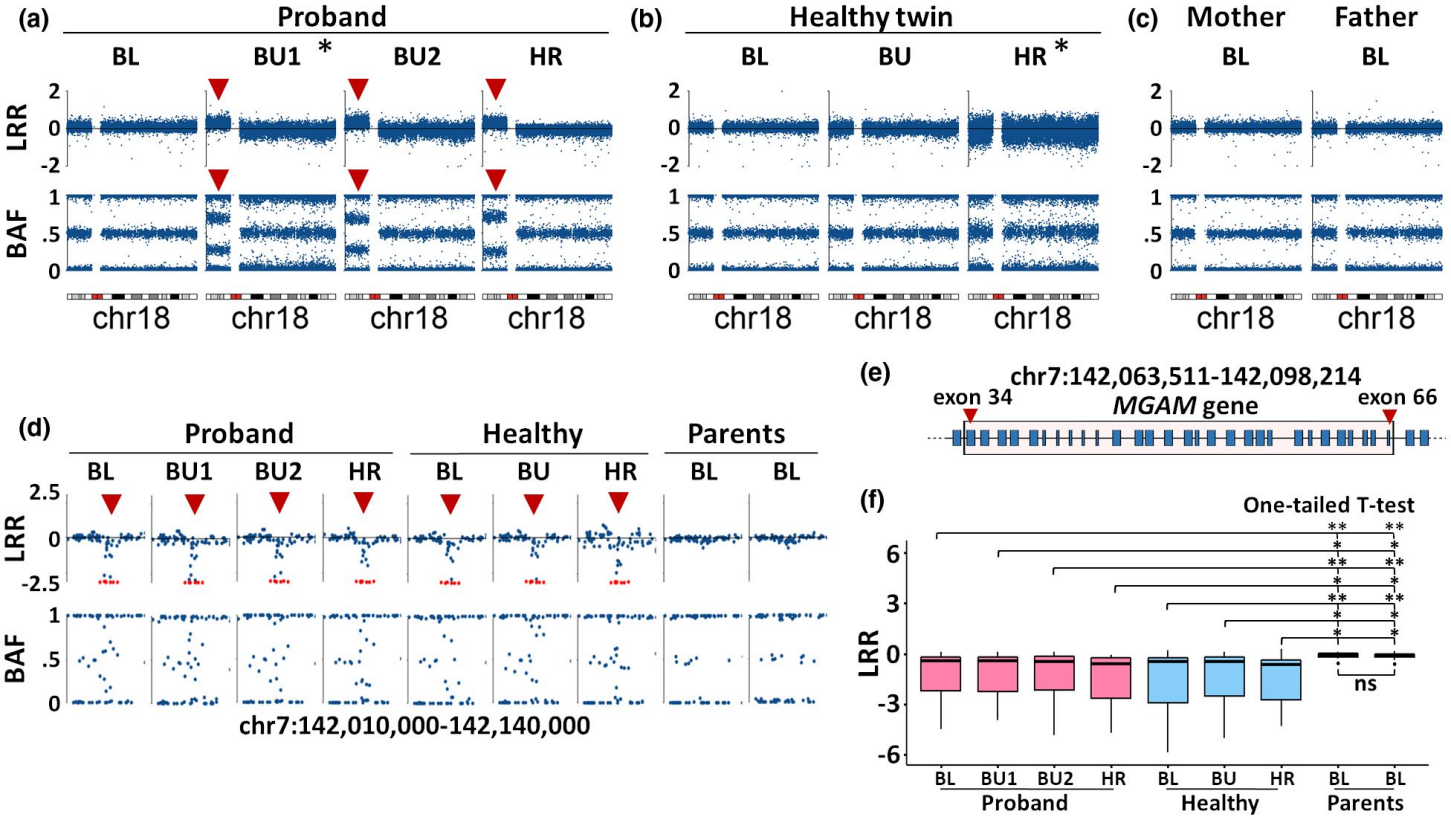
Genome‐wide copy number analysis using Illumina Global Screening Array. Panels (a–c); Log R ratio (LRR; upper part) and B allele frequency (BAF; lower part) plots for markers located on chromosome 18, for the proband (a), phenotypically normal twin (b) and parents (c). Red arrowheads mark copy number change and BAF deviation for markers located in the 18p region. Two experiments marked with an asterisk had a lower quality (genome‐wide Standard Deviation of LRR‐values were >0.28, but fulfilling other quality criteria, such as the SNP call rate for all samples was >98%; and the LogRdev value was <0.2). Panel (d) LRR and BAF plots of 130 kb chromosome 7 region. Red dots denote markers with LRR values below −2.5. Red arrowheads mark the deletion region. Tissue acronyms: BL, blood; BU, buccal; HR, hair follicles. (e) Schematic representation of annotations in chromosome 7. Margins of deletion are determined by SNP markers rs185053832 and rs7801560 and the deletion is approximately 34.7 kb. Panel (e) Red rectangle marks exons in the *MGAM* gene covered by the deletion and red arrowheads mark the positions of exons 34 and 66. Panel (f) Boxplot of LRR values for markers in chromosome 7 deletion region. Statistical significance was tested with one‐tailed t‐test. Results are presented as symbols for pairwise comparisons between each sample from twins versus mother or father. Significance levels: ns, not significant; *p < 0.05; **p < 0.01

**FIGURE 3:**
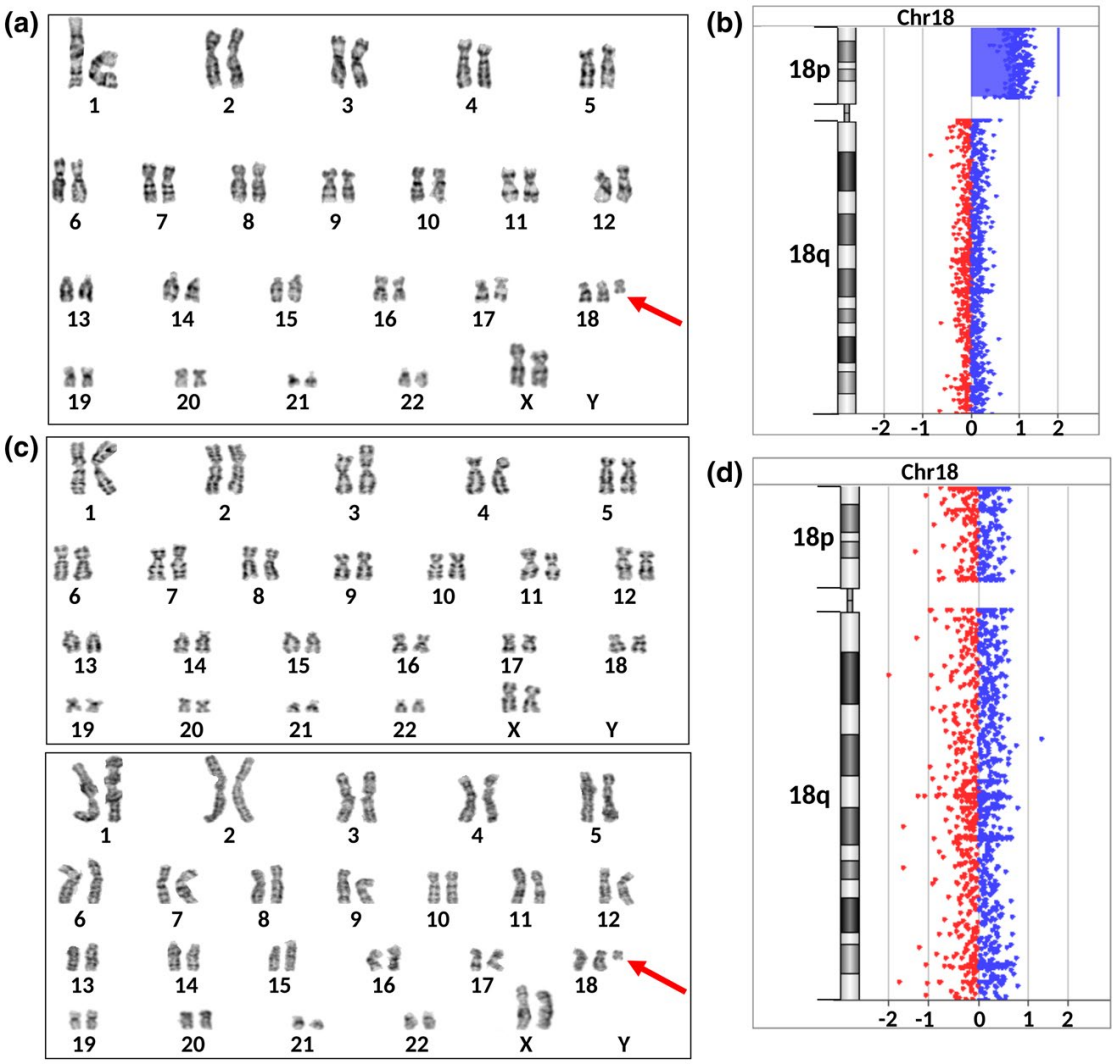
Cytogenetic and molecular findings in cultured skin fibroblasts of PDMZTs. (a) Phenotypically abnormal twin's karyotype 47,XX,i(18)(p10), an arrow indicates supernumerary i(18p). (b) Results of aCGH analysis in proband pointing a gain (four copies) of genetic material at 18p: 18p11.32p11.21(148963_14081887)x4. (c) Karyotypes of phenotypically normal twin: upper panel—normal karyotype 46,XX observed in 69 metaphases, lower panel—abnormal karyotype 47,XX,+i(18)(p10) observe in four metaphases; an arrow indicates i(18p). The presence of two different cell lines indicates mosaic karyotype mos47,XX,+i(18)(p10)[4]/46,XX [69]. (d) Results of array‐CGH analysis in phenotypically normal twin pointing a normal copy number of 18p

In the phenotypically abnormal twin, results from five different experiments in three tissues showed high‐level of mosaicism, consistent with all studied cells being affected by tetrasomy of 18p (Table 1, Figures 2 and 3, Figure S1). Two tissues (fibroblasts and hair follicles) were studied by two methods (array CGH and karyotyping as well as WES and SNP‐array, respectively) and the results were fully concordant. Furthermore, karyotyping of cultured fibroblasts revealed a supernumerary marker chromosome defined as isochromosome 18p [i(18p)]. In the proband, all metaphases (*n* = 60) presented 47,XX,+i(18)(p10) karyotype (Figure 3a). Surprisingly, leukocyte‐derived DNA used for SNP‐array was negative for mosaicism of 18p markers, suggesting that not all tissues in the phenotypically abnormal twin contain i(18p) (Table 1, Figure 2a). The SNP‐array experiment on blood DNA was of high quality (Standard Deviation of Log R Ratio (LRR)‐values for chromosome 1 = 0.13) and if the mosaicism above 5% cells containing 18p gain was present in this sample, it would likely be detected (Conlin et al., 2010b). However, a lower (than ~5%) level of mosaicism in blood is not currently possible to rule out. The high‐level mosaicism in hair follicles and buccal mucosa was clearly visible using both LogR Ratio (LRR) and B Allele Frequency (BAF) values and was consistent with >95% of cells from hair follicles and buccal mucosa of the proband.

**Table 1:** Comparison of mosaicism level of tetrasomy 18p detected in phenotypically discordant monozygotic twins in different cell types and by different analytical methods

Individual	Tested tissue	SNP array	aCGH	WES	Karyotyping
Phenotypically abnormal twin	blood	Normal karyotype or LLM of i(18)p (≤5%)^a^	Not tested	Not tested	Not test
	buccal mucosa # 1	**Full i(18p) or HLM (≥95%)** ^b^	Not tested	Not tested	Not tested
	buccal mucosa # 2	**Full i(18p) or HLM (≥95%)** ^b^	Not tested	Not tested	Not tested
	hair follicles	**Full i(18p) or HLM (≥95%)** ^b^	Not tested	**Full i(18p) or HLM (≥75%)** ^b^	Not tested
	skin fibroblasts	Not tested	**Full i(18p) or HLM (≥90%)** ^b^	Not tested	**Full i(18p): 47,XX,+i(18)(p10) [60] – 100%**
Phenotypically normal twin sister	blood	Normal karyotype or LLM of i(18)p (≤5%)^a^	Not tested	Not tested	Not tested
	buccal mucosa # 1	Normal karyotype or LLM of i(18)p (≤5%)^a^	Not tested	Not tested	Not tested
	hair follicles	Normal karyotype or LLM of i(18)p (≤5%)^a^	Not tested	Normal karyotype or LLM (≤25%)^d^	Not tested
	skin fibroblasts	Not tested	Normal karyotype or LLM of i(18)p (≤10%)c	Not analyzed	**LLM of i(18p): 47,XX,+i(18)(p10) [4]/46XX [69] – 5.4%**
Mother	blood	Normal karyotype or LLM of i(18)p (≤5%)^a^	Not tested	Not tested	Not tested
Father	blood	Normal karyotype or LLM of i(18)p (≤5%)^a^	Not tested	Not tested	Not tested

Identified abnormal karyotype is bolded.

^a^ i(18p) not detected or the presence of low‐level mosaicism (LLM) ≤5%, which is under detection rate for SNP array (Conlin et al., 2010a).

^b^ Full tetrasomy of 18p or the presence of high‐level mosaicism (HLM) ≥95% for SNP array, ≥90% for aCGH and ≥75% for WES.

^c^ i(18p) not detected or the presence of low‐level mosaicism (LLM) ≤10%, which is under detection rate for aCGH (Pham et al., 2014).

^d^ i(18p) not detected or the presence of low‐level mosaicism (LLM) ≤25%, which is under detection rate for WES (King et al., 2017).

### 3.2 The phenotypically normal twin displayed low‐level mosaicism for i(18p) in fibroblasts only

The corresponding analysis performed in the phenotypically normal twin surprisingly revealed low‐level mosaicism for i(18p) in the skin‐derived fibroblasts (Table 1, Figure 3c). Karyotyping of fibroblasts revealed that 4 out of 73 nuclei (5.4%) contained a supernumerary marker chromosome defined as i(18p) {mos 47,XX,+i(18)(p10) [4]/46,XX [69]}.

The result from aCGH experiment using DNA from cultured fibroblasts was negative for the detection of this mosaicism (Table 1, Figure 3d), but this is not surprising, considering that the limit of detection for this method is at least 10% of cells with such an aberration. This low‐level mosaicism in fibroblasts is likely not connected with any detectable clinical phenotype in the phenotypically normal sister.

Whole‐blood DNA of both parents was also analyzed by SNP‐arrays. We found no signs of 18p duplication (Figure 2c). Furthermore, analysis of the opposite homozygotic *loci* in parents and corresponding heterozygotic *loci* in twins revealed that i(18p) was formed by the maternal allele of chromosome 18 (Figure S2).

### 3.3 High level of mosaicism for 7q deletion in both twins

Another aberration uncovered in the twins was a short (34.7 kb) deletion on 7q present in both twins and in all studied tissues, at approximately the same level of the affected cells using SNP‐arrays (Figure 2d–f). Blood DNA from both parents was negative for this deletion. The fact that both twins have this deviation suggests that it is unlikely related to the phenotype of the proband. An inspection of LRR and BAF values for the deleted region suggests that considerably more than 50% of all alleles in the studied samples contain the deletion. This would suggest that it is a bi‐allelic mutation, in at least some of the studied cells from both twins, possibly because of several mutational events. One possible scenario is the occurrence of one mutation in the germline of one of the parents and the second as a post‐zygotic event very early during embryonic development, prior to the twinning have taken place. This 34.7 kb deletion likely inactivates the *MGAM* gene, which encodes maltase‐glucoamylase; enzyme of brush borders of the intestinal lining, playing a role in the final steps of starch digestion. The region containing *MGAM* has been reported as a frequent copy number variation in the Database of Genomic Variants (http://dgv.tcag.ca/).

## 4 DISCUSSION

We describe a pair of PDMZTs where phenotypic features of the proband are due to mosaic tetrasomy 18p, which is preferentially present in the tissues of ectodermal origin. Tetrasomy 18p [or isochromosome 18p, i(18p)] is a rare syndrome (OMIM #614290) involving feeding difficulties, neonatal respiratory distress, growth retardation, microcephaly, developmental delay, muscle tone abnormalities, various congenital anomalies, and characteristic dysmorphism (Sebold et al., 2010; Slimani et al., 2019). Most cases are *de novo* aberrations, although there are familial examples (Abeliovich et al., 1993; Boyle et al., 2001). The phenotypic manifestation differ, with variable presentation especially pronounced in mosaic cases, which are limited in number and mostly described in prenatal circumstances (Bai et al., 2017; Chen et al., 2017; Karimzad Hagh et al., 2017). The largest number of subjects with i(18p) was presented by Sebold et al. (Sebold et al., 2010). The consistent findings were global developmental delay/mental retardation, abnormal muscle tone, brain MRI abnormalities, feeding difficulties, and microcephaly. A similar but milder phenotype is present in the rare cases of high‐level mosaicism (Bai et al., 2017; Slimani et al., 2019), which is consistent with a clinical picture of the proband. The comparison of reported i(18p) phenotypes with our PDMZTs is given in Table S2. Notably, only three cases of low‐level i(18p) mosaicism have been described so far. Abeliovich et al. (1993) reported an adult woman mosaic for i(18p) (3% in peripheral blood) who had slight dysmorphic features but normal intelligence, while her non‐mosaic i(18p) positive daughter was severely affected. Additional two healthy individuals had 9.4% and 5.2% mosaic tetrasomy 18p detected in uncultured urinary cells (Chen et al., 2017). Interestingly, in both cases, prenatal diagnostics revealed low‐level mosaicism in amniocytes (ranging from 5.7% to 33.3%), but not in cord blood (Chen et al., 2012, 2014). In our phenotypically normal twin, we observed low‐level mosaic i(18p) (5.4% of cells) in fibroblasts detected by karyotyping. However, this aberration was undetected by aCGH, which is in agreement with the detection threshold of these methods for chromosomal mosaicism (≥5% (Conlin et al., 2010b), ≥10% (Pham et al., 2014), and ≥25% (King et al., 2017) for Illumina BeadChips, aCGH and WES, respectively).

Genetic mosaicism is defined as the co‐existence of genetically distinct cell populations in an organism derived from a single zygote. The presence of genotype/karyotype in some but not all cells of an individual that occurs through post‐zygotic mutation during or after the first mitotic division of the zygote is called post‐zygotic mosaicism (Forsberg et al., 2017). The timing of mutation occurrence during development strongly influences the proportion of affected cells, their distribution, and phenotypic effects (Campbell et al., 2015). It is even suggested that mitotic chromosomal aberrations in an early blastomere stage may trigger the twinning process (Machin, 1996).

Since mosaic i(18p) was observed in both twins, we conclude that the mutation occurred before the twining process. However, we observe the very unequal distribution of the aberrant cells (100% in phenotypically abnormal twin fibroblasts vs. 5.4% in phenotypically normal twin fibroblasts). Based on the proposed mechanisms of i18p formation (Boyle et al., 2001; Eggermann et al., 1996; Kotzot et al., 1996), mosaic tetrasomy 18p observed in examined twins may be explained by post‐zygotic nondisjunction (47,XX+18), followed by the misdivision of the centromere with the loss of 18q and i(18p) formation in a subset of cells. Thus, two distinct cell lines are present 47,XX,+i(18)(p10) and 46,XX. Alternatively, recent data suggest that the mosaic *de novo* small supernumerary marker chromosomes, including i(18p), may originate through trisomy rescue taking place in the early post‐zygotic cell divisions (Kurtas et al., 2019; Matsubara et al., 2020). It is a multi‐step process beginning with pre‐zygotic maternal meiotic nondisjunction, followed by post‐zygotic partial trisomy rescue by anaphase lagging, incorporation of the supernumerary chromosome into micronucleus that segregates to only one of the two daughter cells, accounting for the mosaic condition with normal cell line and a second one containing the supernumerary chromosome (Kurtas et al., 2019). In partial/uncomplete trisomy rescue, only a part of the original trisomic chromosome is eliminated (18q) while a part remains (18p) and the isochromosome may be formed. In both scenarios, unequal allocation of karyotypically different cells in the blastocyst may lead to different proportions of the two distinct cell populations in each embryo and in consequence to a different mosaicism grade. Other explanation would be bone marrow invasion in utero (microchimerism reported previously for monochorionic dizygotic twins (Aoki et al., 2006)), but it is unlikely since blood cells of the dysmorphic twin were unaffected. However, due to methodology limitations, low‐level mosaicism (<5%) of i(18p) could not be excluded.

Due to the lack of DNA from other tissues than blood, we were not able to exclude mosaicism in the probands’ parents, in particular, in the mother whose chromosome 18 was involved in isochromosome formation. However, only few i(18p) cases were described in which a phenotypically normal mother (Boyle et al., 2001) or mother with slight dysmorphic features (Abeliovich et al., 1993) was expected to be gonadal or gonadosoamtic mosaic, respectively. Given the lack of any phenotypic features in the mother and lack of tetrasomy 18p in blood, we estimate the risk of proband's mother being a mosaic as very low.

The discrepancy between blood (lateral mesoderm) and fibroblasts (paraxial mesoderm) karyotypes of a single individual was previously described in a case of Down, Turner, and Pallister–Killian (tetrasomy of 12p) syndrome patients (Azcona et al., 1999; Choi et al., 2013; Conlin et al., 2012). Interestingly, recently published data suggest that a large fraction of mosaic point mutations that occurred in the first few cell division of the zygote is not detectable in blood cells even if present in different tissue types (including the same germ layers) of the same individual (Muyas et al., 2020) It further supports the hypothesis, that in our PDMZTs i(18p) occurred as a very early‐embryonic aberration.

In conclusion, we emphasize that when mosaicism is suspected, it is recommended to sample multiple tissues, especially in MZTs, since blood micro‐chimerism may mask genetic differences between PDMZTs (Erlich, 2011; Forsberg et al., 2017), and mosaic chromosomal aberrations may be present in blood in fewer cells (or be absent) than in other tissues (Kong et al., 2018). We also highlight the usefulness of non‐invasive sampling of hair follicles and buccal mucosa as a convenient source of non‐blood‐derived DNA of ectodermal origin. Our findings expand evidence that low‐level mosaic i(18p) is well tolerated and does not affect phenotype. Finally, our report shows that low‐level mosaicism, readily detected by classical karyotyping, can be missed by other routinely used methods.

## CONFLICT OF INTEREST

All authors declare no competing interests.

## AUTHORS’ CONTRIBUTIONS

Conception of the study: Rafał Płoski, Jan Dumanski, Małgorzata Rydzanicz, Marco Cavalli, Pawel Olszewski. Sample collection and preparation: Pawel Olszewski, Natalia Filipowicz, Jan Dumanski, Hanna Davies, Dariusz Śladowski, Paweł Krajewski. Planning and preparations of experiments: Małgorzata Rydzanicz, Pawel Olszewski, Natalia Filipowicz, Hanna Davies, Marco Cavalli, Grażyna Kostrzewa, Marlena Młynek. Analysis of the data: Pawel Olszewski, Darek Kedra, Jan Dumanski, Marco Cavalli, Marcin M. Machnicki, Piotr Stawiński, Krystyna Chrzanowska, Rafał Płoski. Collection of clinical data: Krzysztof Szczałuba. Writing of manuscript: Rafał Płoski, Jan Dumanski, Małgorzata Rydzanicz, Pawel Olszewski, Darek Kedra, Hanna Davies, Bozena Bruhn‐Olszewska, Krzysztof Szczałuba. Revisions of the manuscript: All Authors. Funding for the study: Małgorzata Rydzanicz, Jan Dumanski.

## Supporting information

Fig S1‐S2

Table S1‐S2
