# Supplementary material for: Variable degree of mosaicism for tetrasomy 18p in phenotypically discordant monozygotic twins—Diagnostic implications
Source: Mol Genet Genomic Med. 2020 Dec 14;9(1):e1526. doi: 10.1002/mgg3.1526 (PMC7963419; doi:10.1002/mgg3.1526)
Supplement: Supplementary file 1 — Fig S1‐S2 [file MGG3-9-e1526-s001.pdf]

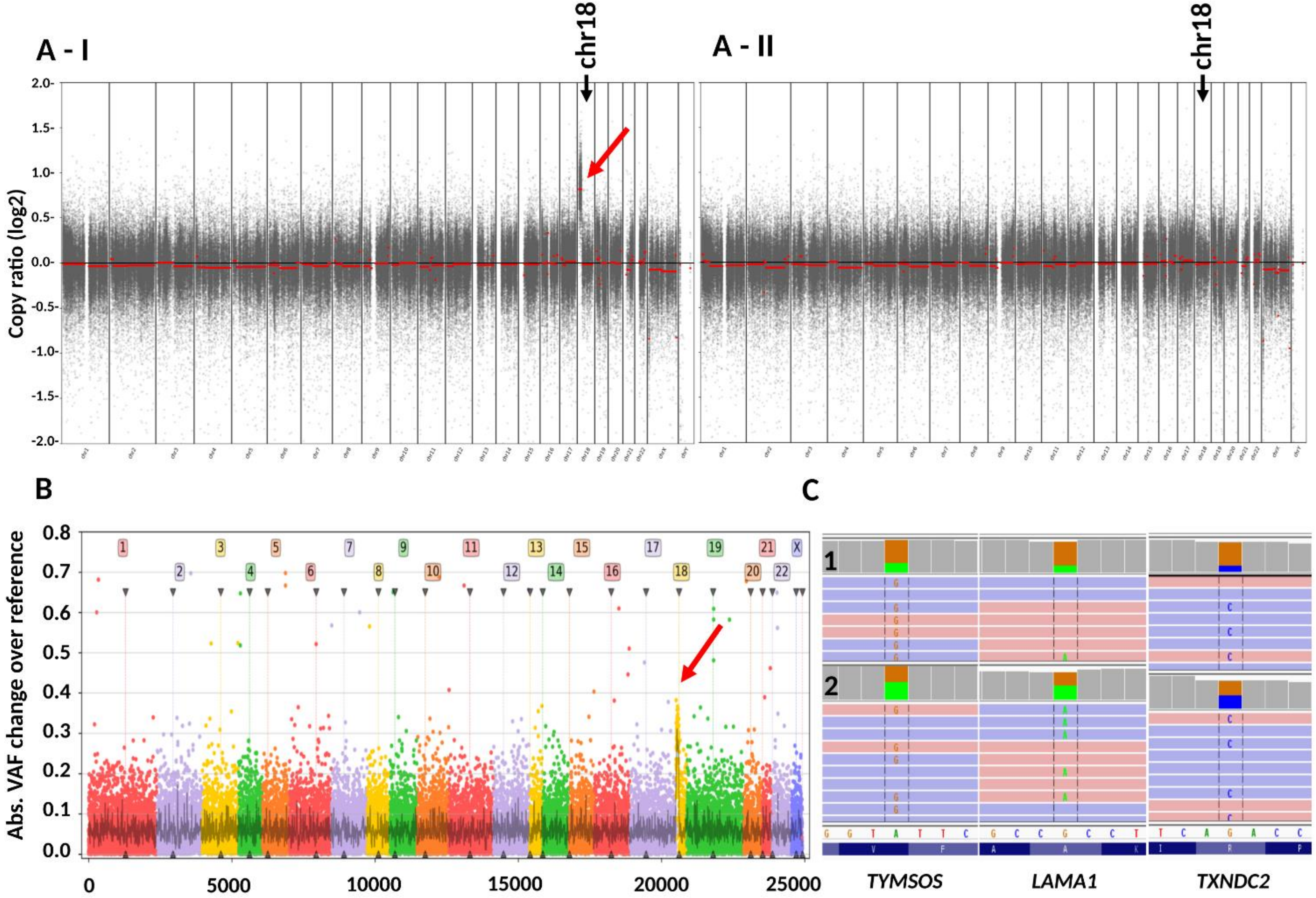

Supplementary Figure 1. **Identification of extra copies of chromosome 18p in phenotypically abnormal twin based on WES data analysis of DNA derived from hair follicles. A** – CNVs analysis: A-I phenotypically abnormal twin, A-II phenotypically normal twin. An arrow indicates a gain of genetic material on chromosome 18p. **B** – comparative analysis of global heterozygosity alterations (phenotypically abnormal twin vs. phenotypically normal twin). Vertical lines on chart B indicate approximate positions of centromeres, broken line represents median values of VAFs, calculated for every 10 consecutive variants. An arrow indicates allelic imbalance. **C** – an IGV screen shots of SNVs located at 18p illustrating an allelic imbalance in phenotypically abnormal twin (1) but not in phenotypically normal twin sister (2). In phenotypically abnormal twin (1) the fraction of mutated/reference alleles were 0.25-0.3 or 0.7-0.75, while in phenotypically normal twin sister (2) were present in heterozygous state (nearly 0.5, 1:1 ratio).

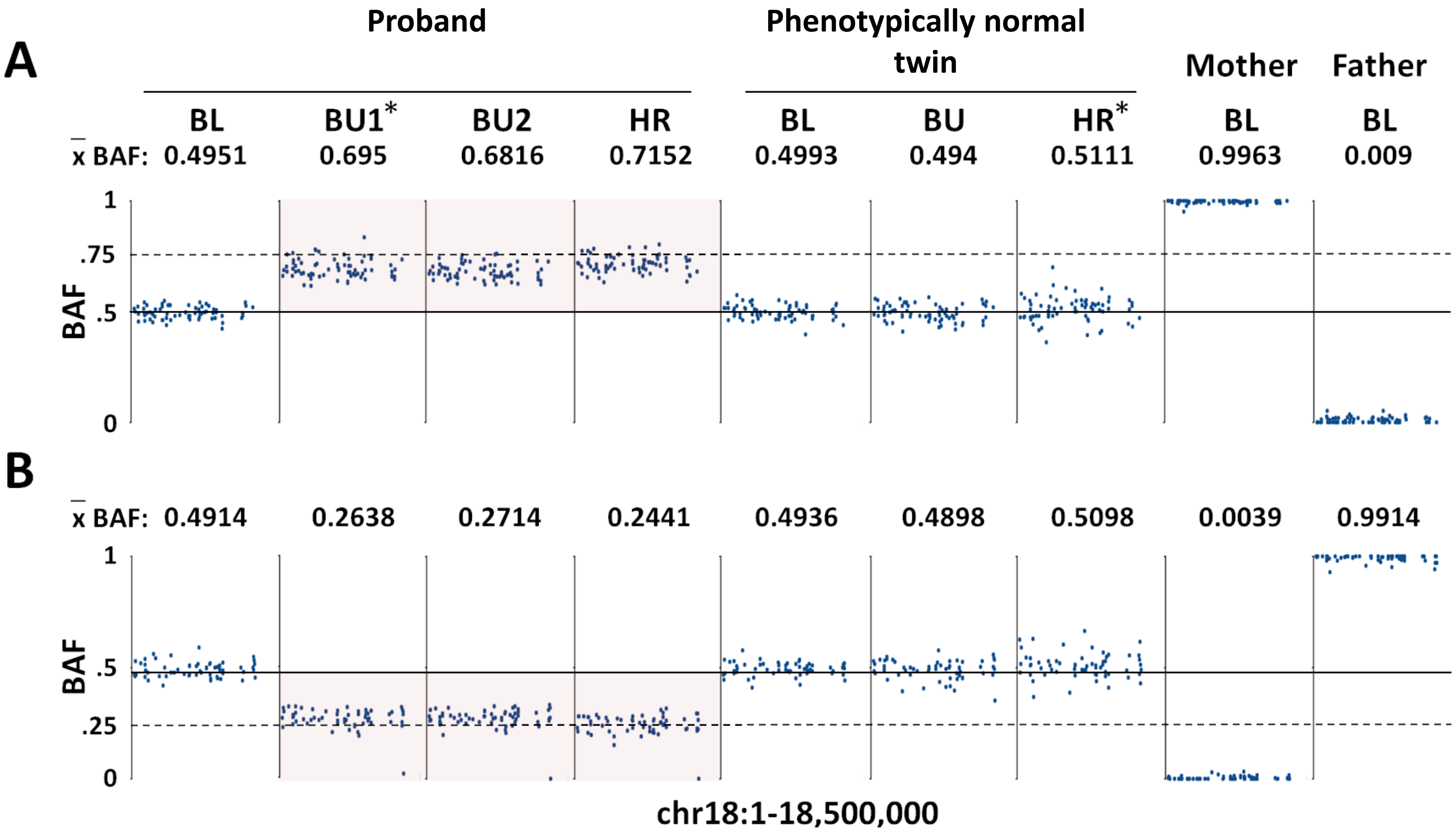

Supplementary Figure 2. Isochromosome 18p is formed from maternal chromosome 18. Vertical columns represent nine Illumina SNP-array experiments in four tissues of the proband, three tissues of the phenotypically normal twin as well as from blood from father and mother. Tissue acronyms: BL - blood, BU - buccal, HR – hair follicles. There were two selection criteria for markers that are shown: i) derived only from 18p (chr18:1-18,500,000); and ii) based on parental values of B allele frequency (BAF), were homozygous in the father and the mother in opposite direction of allele combinations. **Panel A** shows results for selected SNP markers, for which mother was AA and father was BB. **Panel B** display results for selected markers, for which mother was BB and father was AA. Numbers above each SNP profile specify mean BAF values for selected markers. Dashed horizontal lines indicate 0.75 and 0.25 frequency expected for complete tetrasomy. Pink fields highlight strong bias towards maternal allele frequencies in three experiments from two tissues (buccal mucosa sample 1 and 2, as well as hair) in the proband containing isochromosome 18p. Two experiments (marked with an asterisk) had a lower quality (genome-wide Standard Deviation of LRR-values were >0.28, but fulfilling other quality criteria, such as the SNP call rate for all samples was >98%; and the LogRdev value was <0.2).
