# Supplementary material for: Variable degree of mosaicism for tetrasomy 18p in phenotypically discordant monozygotic twins—Diagnostic implications
Source: Mol Genet Genomic Med. 2020 Dec 14;9(1):e1526. doi: 10.1002/mgg3.1526 (PMC7963419; doi:10.1002/mgg3.1526)
Supplement: Supplementary file 2 — Table S1‐S2 [file MGG3-9-e1526-s002.pdf]

SUPPLEMENTARY MATERIALS

Proband's clinical presentation

Supplementary Table 1. Clinical characteristics of the phenotypically abnormal twin with mosaic tetrasomy 18p

Developmental period	Clinical features
Prenatal	No abnormalities detected on US
At birth	Smaller than unaffected twin
Neonatal	Respiratory support<br>Heart rhythm disturbances (+very high troponin C)<br>Dysmorphism: smaller head size, attached earlobes, overlapping fingers, valgus feet (distal limb arthrogryposis); Lethargy<br>Hypotonia
Infantile stage and toddler years	Hypotonia<br>Episodes of vomiting after solid foods<br>Developmental delay (including speech)
At age 6 years (last visit)	Clumsy gait<br>Slurred speech<br>Dysmorphism: open mouth, protruding tongue, hirsutism, frontal nevus, four-limb distal arthrogryposis, coarser facial features than unaffected twin, arched eyebrows, flat philtrum, prominent mandible<br>Strabismus<br>Astigmatism
Results of additional investigations	Brain MRI: partial hypoplasia of corpus callosum<br>Muscle biopsy: unspecific mild myopathic changes<br>Bone age: normal<br>Echocardiogram: normal

Methods

DNA extraction and testing of monozygosity of twins

In Warsaw, DNA was extracted from whole blood using the standard salt-out method. DNA from 30 hair follicles was extracted using the DNA IQ™ Casework Pro Kit for Maxwell® 16 (Promega, Madison, WI, USA). In Gdansk, DNA was extracted from 2 ml of whole blood using QIAmp DNA Blood Midi kit according to the manufacturers protocol (Qiagen, Hilden, Germany). Buccal samples were collected and subsequently processed using ORAcollect-Dx kit (DNA Genotek, Ottawa, Canada). The DNA from hair follicles was extracted using an in-house protocol in Uppsala. The hair was cut into smaller pieces and placed in a lysis buffer containing 10 mM EDTA, 10 mM Tris-HCL (pH 7.9), 50 mM NaCl, 1% N-Lauroylsarcosine sodium salt (Sigma), 10 mg/ml proteinase K (Sigma), and 20µl 1 M DTT (Sigma). This mixture was incubated for 3 hours in 50°C under vigorous shaking. The DNA was then precipitated using Sodium Acetate (pH 5.4) and 96% Ethanol, washed with 80% Ethanol and re-suspended in water. DNA quality and concentration was assessed with TapeStation using Genomic DNA Screen Tape (Agilent, Santa Clara, CA, USA) and PicoGreen reagent (Thermo Fisher Scientific, Waltham, MA, USA).

The monozygosity of twins was determined using two independent methods. DNA from hair follicles was analyzed with AmpFLSTR® NGM™ PCR Amplification Kit (Applied Biosystems, Foster City, USA) composed of 17 highly polymorphic markers. PCR products were separated on 31300xL Genetic Analyzer capillary sequencer (Applied Biosystems, Foster City, CA, USA) and evaluated using GeneMapper ID v3.2.1 (Applied Biosystems). Obtained results were analyzed according to allelic ladder standards. The second method for monozygosity test was pairwise comparisons of genotypic concordance between samples from the family using genotypes of Illumina Global Screening Array-24 v2.0 BeadChip, containing >654,000 markers. An in house developed R script was used to assess genotype similarity between samples. A matrix of B Allele Frequency (BAF) was created from all files, filtered to remove non-existing values (NAs) and used to calculate pairwise Pearson's correlation coefficients between all samples.

Whole exome sequencing (WES) of hair follicles - Single nucleotide variants and in-del analysis

Libraries were prepared using 50 ng of genomic DNA extracted from hair follicles using the SureSelectQXT Reagent Kit and SureSelectXT Human All Exon v5 (Agilent Technologies,

Cedar Creek, TX, USA) and paired-end sequenced (2x100 bp) on HiSeq1500 (Illumina, San Diego, CA, USA). Both samples were sequenced to the mean coverage of >100x; in affected twin 95% of the exome was covered at minimum 20x, and 98% was covered at least 10x. Corresponding numbers for the unaffected twin were 94% and 97%, respectively. WES data were analyzed as previously described (Rydzanicz et al., 2019) in agreement with the Broad Institute recommendations (https://software.broadinstitute.org/gatk/best-practices/). In brief, raw data was analyzed with bcl2fastq software (Illumina, San Diego, CA, USA) to generate reads in fastq format. After the quality control step, including adapter trimming and low quality reads removal, reads were aligned to the GRCh37 (hg19) reference genome with Burrows-Wheeler Alignment Tool (http://bio-bwa.sourceforge.net/) and processed further by Picard (http://broadinstitute.github.io/picard/) and Genome Analysis Toolkit (https://software.broadinstitute.org/gatk/). In particular, base quality score recalibration, indel realignment and duplicate removal were executed, and the SNVs and INDELs discovery was performed. Identified variants were further annotated with functional information, frequency in population (including EXaC, gnomAD, dbSNP, dbNSFP, 1000 genomes, as well as the frequency from in-house database of >3000 Polish individuals screened by WES), and known association with clinical phenotypes based on both ClinVar (Landrum et al., 2014) and HGMD (Stenson et al., 2003). *In silico* pathogenicity prediction was performed bade on Varsome (https://varsome.com) pathogenicity and conservation scores. Additionally, for enhancing the sensitivity of low allele fraction (possible mosaic state) GATK MuTect2 was used (Cibulskis et al., 2013); phenotypically abnormal twin was treated as “cancer”, whereas phenotypically normal twin as “normal” sample. Rare (frequency <0.01 in all tested databases) functional variants in protein-coding regions (missense, frameshift, stop-loss, stop gain) and variants in splicing regions were considered. Priority was given to variants presented only in phenotypically abnormal twin (while in phenotypically normal co-twin lack of variant in given genomic position was confirmed if covered >20x). All prioritized variants were manually inspected in Integrative Genomics Viewer (Robinson et al., 2011).

Copy Number Variants and Loss of Heterozygosity (LOH) analysis based on WES

Copy-number variation calling was performed using CNVkit 0.9.5 [ref.] (Talevich, Shain, Botton, & Bastian, 2016). A panel of samples with normal karyotype sequenced with the same WES target (Agilent SureSelectXT Human All Exon v5) was used to calculate reference coverage. CNVs were detected by comparing coverages of individual normal (phenotypically

normal twin) and case samples (phenotypically abnormal twin) with the computed reference coverage. Absolute copy numbers were called using default CNVkit thresholds.
Global LOH analysis was performed using an in-house python script (employing pandas and matplotlib libraries), comparing normal and case samples. SNVs (hg19) identified by GATK HaplotypeCaller (Van der Auwera et al., 2013) with allele frequency >0.001 in ExAC (Lek et al., 2016) or 1000 genomes (Genomes Project et al., 2015) databases, ≥20x coverage in the normal sample and with variant allele frequencies (VAF) in range (0.3, 0.7) were selected for analysis. VAFs of corresponding variants were subtracted to calculate absolute VAF shift between phenotypically abnormal and phenotypically normal twin sisters. Approximate boundaries of regions with LOH were calculated using circular binary segmentation algorithm implemented into CNVkit.

Cytogenetic analysis

Karyotype studies were performed from standard cultured fibroblasts from skin biopsies, followed by G-banding technique. A 400-band resolution metaphase chromosomes were analyzed for each patient using Cytovision® Karyotyping software version 7.4. Genomic DNA was isolated from fibroblast culture using a Sherlock AX kit (A&A Biotechnology), according to the manufacturer's protocol.

Array-CGH analysis of DNA extracted from skin fibroblasts

The whole genome array-CGH procedure was performed following the manufacturer's instructions (SurePrint G3 ISCA V2 CGH Microarray Kit; Agilent Technologies). The 60K slides were scanned on a NimbleGen 200 Microarray Scanner (Roche). Feature extraction and data analysis was carried out with Agilent CytoGenomics 5.0.2.5 software (Agilent Technologies) using default analysis settings. The aCGH results were analyzed with the UCSC hg19 assembly.

Genotyping with Illumina SNP beadchips and calling of copy number variants

We performed the SNP genotyping using Global Screening Array-24 Kit v2.0
according to the recommendations of the manufacturer at the SNP-Seq Technology Platform (SciLifeLab, Uppsala University, Sweden). Routine quality control measurements were performed as recommended by Illumina. The SNP call rate for all samples was >98%; and the LogRdev value was <0.2. An additional criterion recommended by Illumina for studies of

copy number variation was fulfilled such as genome-wide Standard Deviation of log R ratio (LRR)-values below 0.28 (www.illumina.com/content/dam/illuminamarketing/ documents/products/appnotes/appnote_cnv_loh.pdf). Two experiments (buccal mucosa sample 1 from the proband and hair from the phenotypically normal twin) had somewhat lower quality; genome-wide Standard Deviation of LRR-values were >0.28, but fulfilling other quality criteria, such as the SNP call rate for all samples was >98%; and the LogRdev value was <0.2. The results from Illumina SNP arrays consist of two main data tracks: LRR and B-allele frequency (BAF). Deviations of consecutive probes from normal states are indicative of structural genomic aberrations. We analyzed Illumina output files by using Nexus Copy Number 10.0 software (BioDiscovery, CA, USA), which is applying the ‘‘SNP-FASST2 Segmentation’’ algorithm. It is a Hidden Markov Model based algorithm in which LRR and BAF values are included in the segmentation process, generating both copy-number and allelic-imbalance calls, as previously described (Forsberg et al., 2012).

Results

WES single nucleotide variant (SNVs)/indel analysis (hair follicles)

For both samples over 55 million read pairs were generated resulting in average mean depth >100x, coverage min. 10x > 97% and min. 20x > 94x. Total number of variants identified in proband was 128,841, whereas in phenotypically normal twin was 127,407. The 1,434 possibly discordant variants were manually reviewed. However, all were classified as false-positive, mostly due to low coverage or location within repetitive sequence.

Supplementary Table 2. Comparison of the phenotypic features reported for individuals with full tetrasomy 18p, postnatal mosaic tetrasomy 18p, and the PDMZTs reported in the present study.

**Phenotypic features**	**Full tetrasomy 18p**†	**Mosaic tetrasomy 18p**‡	**Our PDMZTs**
Facial dysmorphism	39/39 (100%)§	1/2	1/2
Developmental delay/ Mental retardation	114/115 (99%)	2/2	1/2
Abnormal muscle tone	80/115 (69%)	0/2	1/2
Neonatal complications	69/107 (64%)(data not given in ref. 12)	1/2	1/2
Microcephaly	58/104 (56%)	2/2	1/2
Brain MRI anomalies	11/22 (50%)	0/2	1/2
Strabismus	43/97 (44%)	1/2	1/2
Scoliosis/kyphosis	31/84 (37%) (data not given in ref. 12)	0/2	0/2
Growth retardation	34/104 (33%)	1/2	0/2
Recurrent otitis media	37/115 (32%)	0/2	0/2
Constipation	34/107 (32%)	0/2	0/2
Heart defect	26/104 (25%)	1/2	0/2
Foot/Hand anomalies	21/92 (23%) (feet only)	2/2	1/2
Twisting of extremities	0/115	0/2	1/2

† For the full tetrasomy 18p cases the data summed up by Sebold et al., (Sebold et al., 2010) from their study as well as the previous ones has been combined with eight cases described by Slimani et al., (Slimani et al., 2019) (‘data not given in ref. 12’ means that a certain feature was not described by Slimani et al.).

‡ For mosaic tetrasomy 18p a single case by Slimani et al., (Slimani et al., 2019) and a single case by Bai et al., (Bai et al., 2017) were included.

§ number of patients presenting given phenotypic features / total number of examined patients

References:

Bai, J. L., Jin, Y. W., Qu, Y. J., Wang, H., Cao, Y. Y., & Song, F. (2017). Mosaicism of Tetrasomy 18p: Clinical and Cytogenetic Findings in a Female Child. *Chin Med J (Engl), 130*(6), 744-746. doi:10.4103/0366-6999.201604

Cibulskis, K., Lawrence, M. S., Carter, S. L., Sivachenko, A., Jaffe, D., Sougnez, C., . . . Getz, G. (2013). Sensitive detection of somatic point mutations in impure and heterogeneous cancer samples. *Nat Biotechnol, 31*(3), 213-219. doi:10.1038/nbt.2514

Forsberg, L. A., Rasi, C., Razzaghian, H. R., Pakalapati, G., Waite, L., Thilbeault, K. S., . . . Dumanski, J. P. (2012). Age-related somatic structural changes in the nuclear genome of human blood cells. *Am J Hum Genet, 90*(2), 217-228. doi:10.1016/j.ajhg.2011.12.009

Genomes Project, C., Auton, A., Brooks, L. D., Durbin, R. M., Garrison, E. P., Kang, H. M., . . . Abecasis, G. R. (2015). A global reference for human genetic variation. *Nature, 526*(7571), 68-74. doi:10.1038/nature15393

Landrum, M. J., Lee, J. M., Riley, G. R., Jang, W., Rubinstein, W. S., Church, D. M., & Maglott, D. R. (2014). ClinVar: public archive of relationships among sequence variation and human phenotype. *Nucleic Acids Res, 42*(Database issue), D980-985. doi:10.1093/nar/gkt1113

Lek, M., Karczewski, K. J., Minikel, E. V., Samocha, K. E., Banks, E., Fennell, T., . . . Exome Aggregation, C. (2016). Analysis of protein-coding genetic variation in 60,706 humans. *Nature, 536*(7616), 285-291. doi:10.1038/nature19057

Robinson, J. T., Thorvaldsdottir, H., Winckler, W., Guttman, M., Lander, E. S., Getz, G., & Mesirov, J. P. (2011). Integrative genomics viewer. *Nat Biotechnol, 29*(1), 24-26. doi:10.1038/nbt.1754

Rydzanicz, M., Wachowska, M., Cook, E. C., Lisowski, P., Kuzniewska, B., Szymanska, K., . . . Ploski, R. (2019). Novel calcineurin A (PPP3CA) variant associated with epilepsy, constitutive enzyme activation and downregulation of protein expression. *Eur J Hum Genet, 27*(1), 61-69. doi:10.1038/s41431-018-0254-8

Sebold, C., Roeder, E., Zimmerman, M., Soileau, B., Heard, P., Carter, E., . . . Cody, J. D. (2010). Tetrasomy 18p: report of the molecular and clinical findings of 43 individuals. *Am J Med Genet A, 152A*(9), 2164-2172. doi:10.1002/ajmg.a.33597

Slimani, W., Ben Khelifa, H., Dimassi, S., Chioukh, F. Z., Jelloul, A., Kammoun, M., . . . Mougou-Zerelli, S. (2019). Clinical and molecular findings in nine new cases of

tetrasomy 18p syndrome: FISH and array CGH characterization. *Mol Cytogenet, 12*, 5. doi:10.1186/s13039-019-0414-8

Stenson, P. D., Ball, E. V., Mort, M., Phillips, A. D., Shiel, J. A., Thomas, N. S., . . . Cooper, D. N. (2003). Human Gene Mutation Database (HGMD): 2003 update. *Hum Mutat, 21*(6), 577-581. doi:10.1002/humu.10212

Talevich, E., Shain, A. H., Botton, T., & Bastian, B. C. (2016). CNVkit: Genome-Wide Copy Number Detection and Visualization from Targeted DNA Sequencing. *PLoS Comput Biol, 12*(4), e1004873. doi:10.1371/journal.pcbi.1004873

Van der Auwera, G. A., Carneiro, M. O., Hartl, C., Poplin, R., Del Angel, G., Levy-Moonshine, A., . . . DePristo, M. A. (2013). From FastQ data to high confidence variant calls: the Genome Analysis Toolkit best practices pipeline. *Curr Protoc Bioinformatics, 43*, 11 10 11-33. doi:10.1002/0471250953.bi1110s43
